# Optic nerve head abnormalities in primary open-angle glaucoma and their associations with axial length

**DOI:** 10.1007/s10384-026-01371-y

**Published:** 2026-05-08

**Authors:** Hitomi Saito, Weiwei Wang, Kaho Akiyama, Shuichiro Aoki, Shiroaki Shirato, Rei Sakata, Makoto Aihara, Megumi Honjo

**Affiliations:** 1 Department of Ophthalmology, University of Tokyo Graduate School of Medicine, 7-3-1 Hongo, Bunkyo-ku, Tokyo 113-8655 Japan; 2 Department of Ophthalmology, Shaanxi Eye Hospital, Xi’an People’s Hospital (Xi’an Fourth Hospital), Affiliated People’s Hospital, Northwest University, Xi’an, 710004 China; 3 Yotsuya Shirato Eye Clinic, 2-6 Samoncho, Shinjuku-ku, Tokyo Japan

**Keywords:** Lamina cribrosa defect, Prelaminar schisis, Peripapillary retinoschisis, Peripapillary intrachoroidal cavitation, Myopia

## Abstract

**Purpose:**

To determine the frequency of peripapillary retinoschisis (PRS), lamina cribrosa defect (LCD), peripapillary intrachoroidal cavitation (PICC), and prelaminar schisis (PLS) in primary open-angle glaucoma (POAG) eyes and investigate their association with axial length (AXL).

**Study design:**

Retrospective cross-sectional

**Methods:**

Optical coherence tomography scans of 263 POAG eyes were reviewed for PRS, LCD, PICC, and PLS. The frequency of each optic nerve head (ONH) abnormality was compared between eyes with and without high myopia (HM), defined as AXL > 26 mm. Associations with AXL were assessed using univariable and multivariable logistic regression, adjusting for gender, age, intraocular pressure, visual field mean deviation, circumpapillary retinal nerve fiber layer thickness and Bruch’s membrane opening (BMO) area.

**Results:**

PRS, LCD, PICC, and PLS were observed in 11.8%, 31.6%, 18.3%, and 55.1% of all POAG eyes, respectively. 77.2% exhibited at least one abnormality, and 31.2% had multiple abnormalities. PRS, LCD, and PICC were more frequent in HM eyes (*p* = 0.001, 0.016 and <0.001, respectively) and associated with longer AXL in univariable analysis (*p* < 0.001). Multivariable analysis showed that PRS significantly associated with longer AXL (*p* < 0.001) and that LCD and PICC significantly associated with larger BMO area (*p* = 0.009, < 0.001). PLS was more frequent in eyes without HM (*p* = 0.026) and significantly associated with shorter AXL (*p* = 0.005).

**Conclusion:**

ONH abnormality was common in myopic POAG eyes with substantial overlaps. PRS, LCD and PICC were associated with longer AXL and myopic ONH structural change while PLS was associated with shorter AXL. These findings will be a foundation for further studies exploring their functional relevance.

**Supplementary Information:**

The online version contains supplementary material available at https://doi.org/10.1007/s10384-026-01371-y.

## Introduction

High myopia (HM) is a risk factor for primary open-angle glaucoma (POAG) [1, 2]. POAG eyes with HM often exhibit distinct characteristics, such as more pronounced central visual field defects [3–5] and a weaker association between intraocular pressure (IOP) and disease development or progression [6, 7]. Tissue stretching, thinning, and deformation resulting from axial length (AXL) elongation can lead to various structural abnormalities within and around the optic nerve head (ONH). These AXL-associated structural changes are thought to contribute to glaucoma susceptibility or to cause direct functional damage in HM eyes. Therefore, evaluation of abnormalities in the intra-optic disc region including the lamina cribrosa (LC) and peripapillary sclera, holds a key role in identifying structural changes that may underlie functional impairment in myopia and glaucoma.

Advances in optical coherence tomography (OCT) have enabled detailed observation of ONH microstructures. Several ONH abnormalities, including peripapillary retinoschisis (PRS) [8–11], LC defects (LCD) [8, 12–16], peripapillary intrachoroidal cavitation (PICC) [8, 17, 18], and prelaminar schisis (PLS) [19–22] have been identified on OCT images of myopic and glaucomatous eyes. Previous studies report a high prevalence of ONH abnormality in pathologically myopic (78.7%) [23] and non-pathological HM eyes (91.86%) [8], with 49.4%-68.6% coexistence. Although association between longer AXL and these ONH abnormalities has been suggested within these cohorts of HM eyes, whether they are truly associated with longer AXL in POAG remains unclear. Thus, a comprehensive study is needed to explore the frequency, overlap, and relationship with AXL of these ONH abnormalities in a broader POAG population.

The purpose of this study was to determine the frequency and coexistence of PRS, LCD, PICC, and PLS in a cohort of POAG eyes with and without HM. We further investigated the relationship between AXL and these ONH abnormalities to evaluate the influence of myopia on their presentation in POAG.

## Subjects and methods

Protocols for this retrospective cross-sectional study were approved by the Japan Medical Association Ethics Review Committee (R6-19) and adhered to the tenets of the Declaration of Helsinki. The need for written informed consent was waived due to the retrospective nature of the study.

### Subjects

**Fig. 1:**
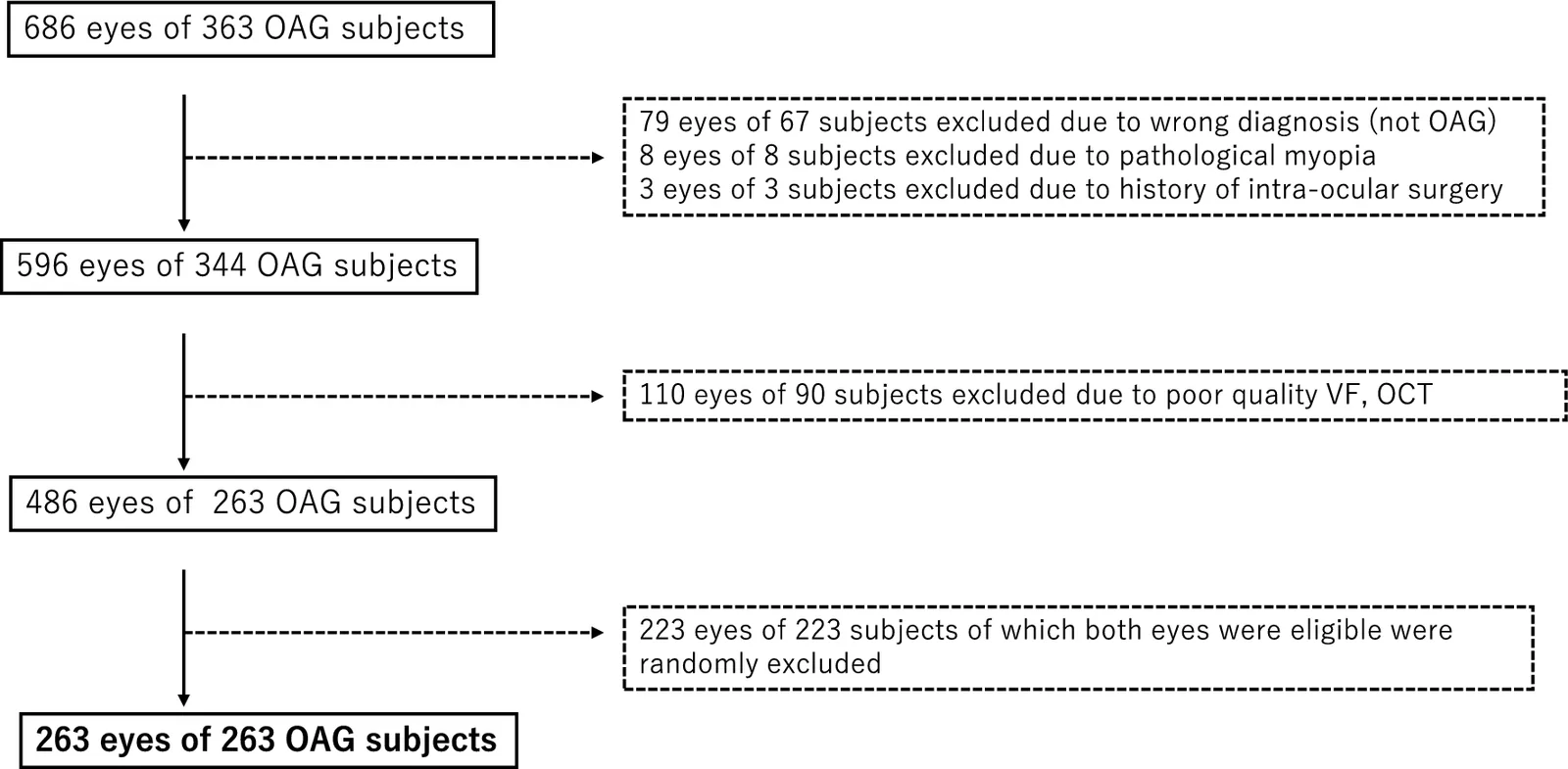
Subject inclusion and exclusion flowchart. *OAG* open angle glaucoma, *VF* visual field, *OCT* optical coherence tomography

We reviewed medical records of all subjects who had visited the Yotsuya Shirato Eye Clinic (Tokyo, Japan) between February 2023 and July 2023 and consecutively enrolled previously diagnosed POAG subjects for whom OCT scans (ONH-centered radial scans and ONH cube scans), visual field (VF) tests, and AXL measurements were available. Exclusion criteria were: 1) glaucoma subtypes other than POAG; 2) age under 20 years; 3) history of prior ocular surgery excluding routine cataract surgery; 4) poor OCT imaging quality (signal strength index < 8); 5) unreliable VF results (fixation losses > 20%, false positives > 15%, or false negatives > 15%); 6) pathologically myopic eyes (defined by the presence of diffuse chorioretinal atrophy, patchy chorioretinal atrophy, macular atrophy, lacquer cracks, myopic choroidal neovascularization, Fuchs’ spot, or posterior staphyloma); 7) non-glaucomatous ocular diseases; and 8) inability to identify ONH abnormalities due to obscured visualization of the temporal ONH region on OCT radial scans. When both eyes of a subject met the inclusion criteria, one eye was randomly selected for analysis. A flow chart detailing subject inclusion and exclusion is presented in Figure 1.

All subjects included in this study underwent the following ocular examinations: refraction and corneal curvature radius measurements, central corneal thickness measurements (CEM-530, NIDEK), best corrected visual acuity, AXL measurements (IOL master, Carl Zeiss Meditec), slit-lamp examination, IOP measurements with Goldmann applanation tonometry, gonioscopy, fundus examination including ONH examination, fundus photography (TRC-NW8F, Topcon), OCT imaging (Cirrus HD-6000, Carl Zeiss Meditec) and Humphrey Field Analyzer (HFA) measurements with the 24-2 Swedish Interactive Threshold Algorithm standard strategy (Carl Zeiss Meditec, Dublin) within 3 months of the OCT scanning.

A POAG eye had IOP < 21 mmHg on the day of OCT acquisition, an open angle, glaucomatous optic disc appearance based on clinical stereoscopic examination by two glaucoma specialists (HS, KA) and abnormal HFA results corresponding to the optic glaucomatous changes. Abnormal HFA results were determined following criteria defined by the Hodapp-Parrish-Anderson criteria [24].

### Identification of ONH abnormality

Presence of ONH abnormalities (PRS, LCD, PICC and PLS) was primarily assessed using 12 ONH-centered 6.0 mm radial scans after pupillary dilation using the Cirrus HD-6000 OCT. All evaluations were conducted independently by two glaucoma specialists (HS, WW) who were masked to each other’s diagnosis. Subsequently, all B-scans from the 200 × 200 ONH cube scans were reviewed by the same examiners to confirm abnormalities identified on the radial scans. In cases of disagreement, a final decision was made by discussing the case with a third adjudicator (KA).

**Fig. 2:**
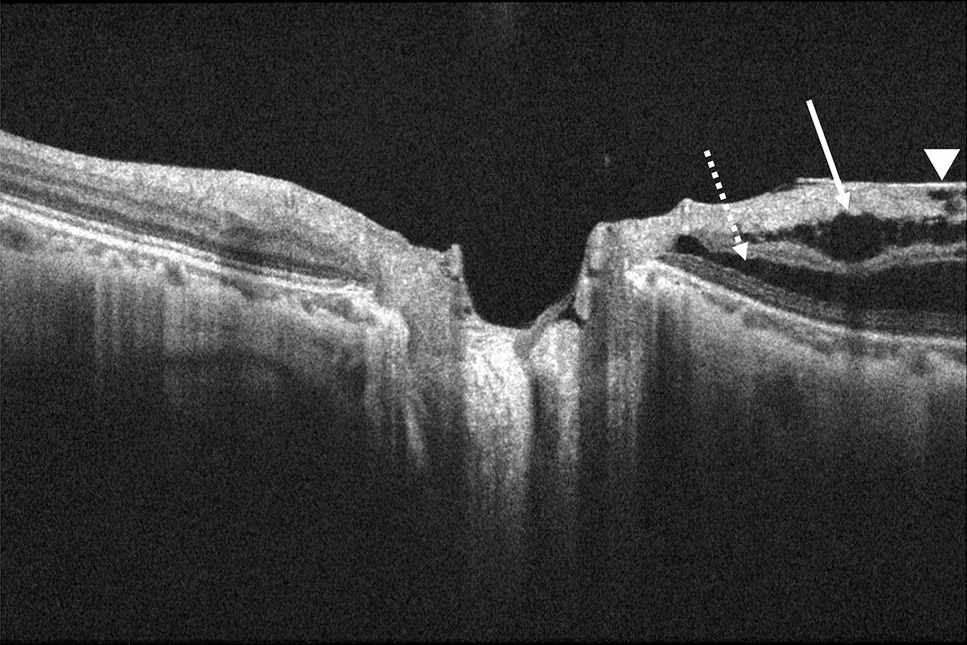
Peripapillary retinoschisis. Splitting of retinal layers is observed in the nerve fiber layer (white arrow head), ganglion cell layer and inner plexiform layer (white arrow) and outer nuclear layer (white dotted arrow)

PRS was defined as splitting of the inner or outer retinal layers in the peripapillary region outside the Bruch’s membrane opening (BMO). (Fig. 2) Schisis isolated to the superficial nerve fiber layer were excluded from analysis due to their minimal size.

**Fig. 3:**
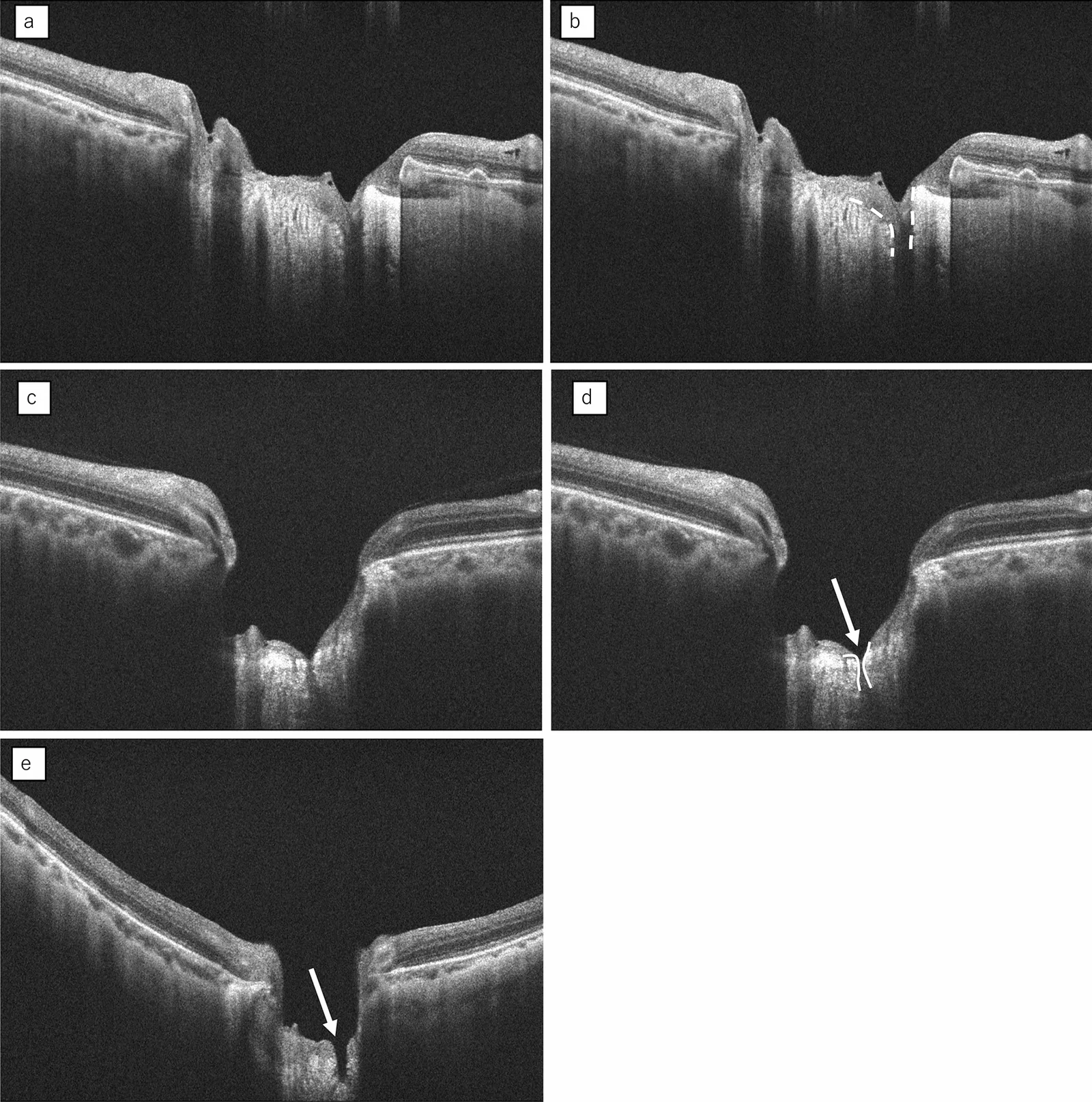
Lamina cribrosa defect. **a & b** Peripheral disinsertion (white dotted line): disruption of the anterior laminar contour in the temporal peripheral region of the lamina cribrosa. **c & d** Central defect (white arrow and white dotted line): disruption in the central region of the lamina cribrosa with visible intact anterior lamina cribrosa surface on both sides of the defect. **e** Acquired pit-like defect (white arrow)

LCD was defined as disruption of the anterior laminar curvilinear U- or W-shaped contour with diameter > 100μm and depth > 30μm in two or more consecutive radial scans [12, 25]. LCD was further sub-classified into peripheral disinsertions characterized by posteriorly displaced laminar insertion with a downward slope at the periphery of the LC toward the neural canal wall, central defects with defects within the BMO where the anterior LC was visible on either side of the defect and acquired pit-like defects. (Fig. 3)

**Fig. 4:**
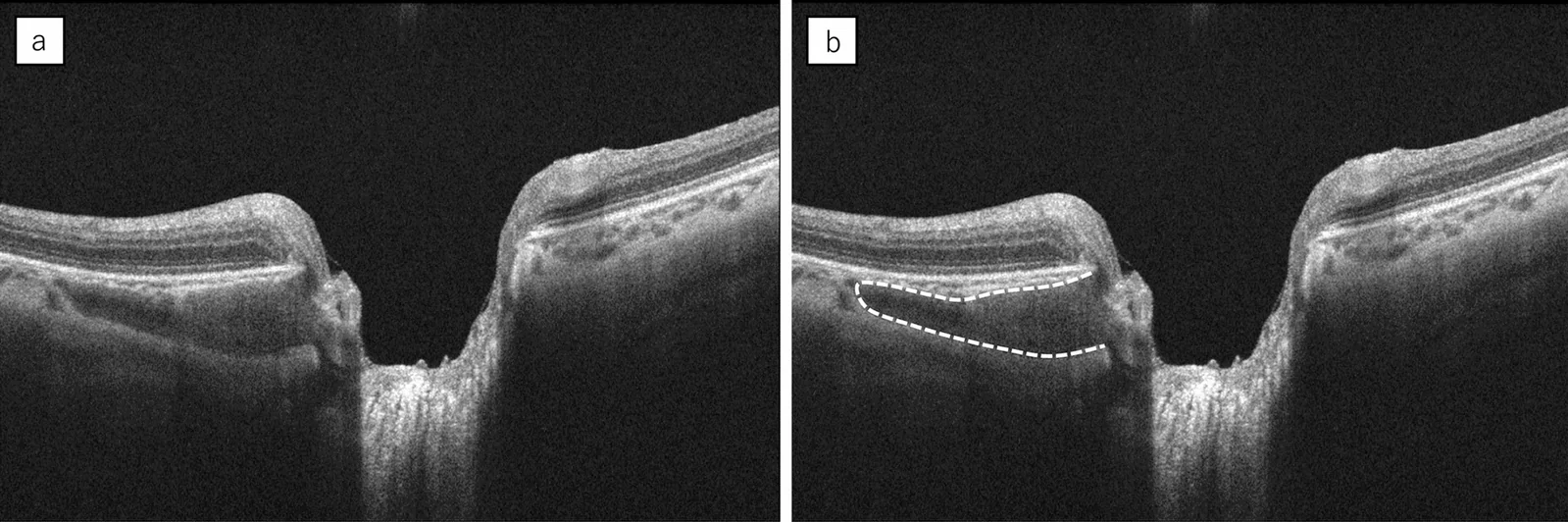
Peripapillary intrachoroidal cavitation. a & b. Triangular hyporeflective areas in the temporal peripapillary choroidal space accompanying posterior peripapillary scleral bowing. (white dotted line)

PICC was defined as triangular hyporeflective areas in the peripapillary choroidal space accompanying posterior peripapillary scleral bowing observed in two or more consecutive radial scans [17, 26] (Fig. 4). Small PICCs with PICC depth < 50μm and tubelike hyporeflective areas without scleral bowing corresponding to choroidal vessels on fundus photographs were excluded from the analysis. PICC depth was calculated as the distance from BMO to the sclera subtracted from the circumpapillary choroidal thickness (cpChT) of the corresponding region to adjust for differences in choroidal thickness among subjects [17].

**Fig. 5:**
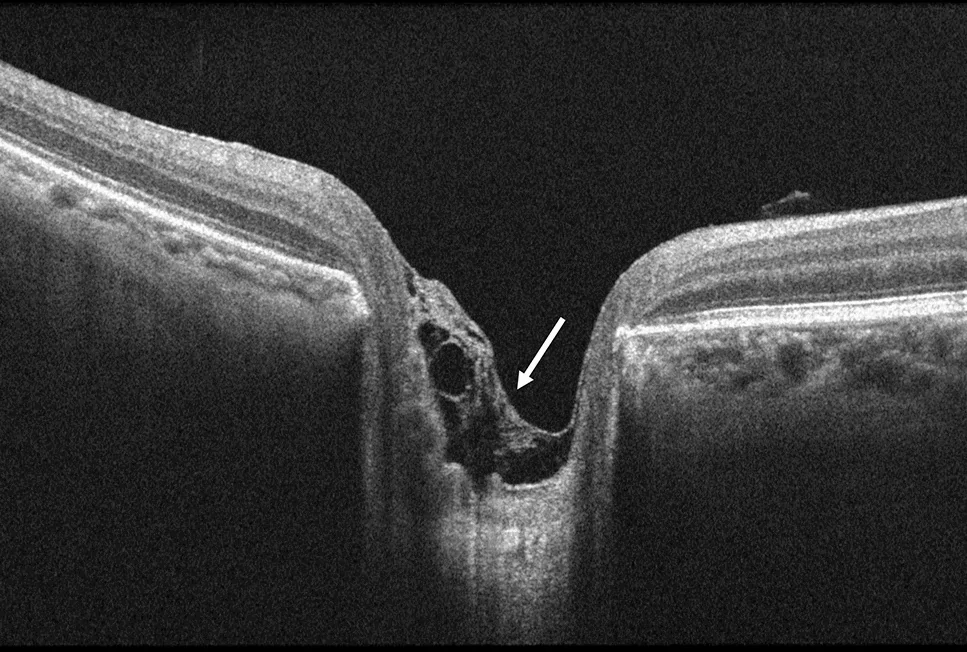
Prelaminar schisis. Prelaminar tissue splitting with detachment of blood vessels together with the intra-disc inner limiting membrane from the underlying prelaminar tissue. (white arrow)

PLS was defined as prelaminar tissue splitting within the BMO boundary in at least two consecutive radial scans [19–22]. (Fig. 5) Subtle schisis with only small superficial optical voids were excluded from analysis.

### OCT Measurements

Global and 6 Garway-Heath sectoral [27] circumpapillary retinal nerve fiber layer thickness (cpRNFLT) and cpChT were measured on 3.46 mm and 4.5 mm diameter BMO-centered measurement circles, respectively. Global cpRNFLT values were calculated from exported ONH cube scan data automatically segmented by custom Zeiss software. CpChT was manually measured on each radial slice using Fiji ImageJ (Java 1.8.0_322 64 bit, National Institutes of Health).

BMO and scleral flange opening (SFO) points were manually determined on both sides of the 12 ONH-centered radial slices by 2 experienced examiners (KA, HS) using Fiji ImageJ. SFO, a recently proposed landmark representing the neural canal opening, is defined as the transition between the anterior laminar surface and the anterior surface of scleral flange on OCT images [17, 28]. The BMO and SFO coordinates were imported into R software version 4.4.1 (R Foundation for Statistical Computin), and a plane minimizing the mean squared axial error was fitted to the 24 BMO and SFO points to determine BMO area and SFO/BMO offset magnitude. The SFO/BMO offset magnitude, representing the degree of SFO/BMO displacement and clinically corresponding to the extent of peripapillary atrophy–gamma and –delta, was defined as the distance between the BMO centroid and the SFO centroid projected onto the BMO plane. (Supplementary Fig. S1) Excellent intra-observer and inter-observer reproducibility of BMO area (intraclass correlation: 0.994 and 0.991, respectively) and SFO/BMO offset magnitude (intraclass correlation: 0.966 and 0.967, respectively) has been previously reported [17].

Measurement circle diameters for cpRNFLT and cpChT, LCD width and planar components of BMO area were corrected for magnification error using the modified Littmann’s formula [29, 30]. The true dimension (*t*) was calculated as *t* = *p* × *q* × *s*, where *s* represents the measured value, *p* is a device-specific constant (3.382), and *q* is an ocular magnification factor derived from AXL (*q* = 0.01306 × [AXL − 1.82]).

### Statistical analysis

Statistical analyses were performed with IBM SPSS statistics (version 27; International Business Machines [IBM] Corp.). POAG eyes were divided into eyes with and without HM at an AXL of 26mm. Differences between eyes with and without HM as well as eyes with and without each ONH abnormality were evaluated using unpaired 2-sided t-tests (or Welch’s t-test) for continuous variables and by Chi-square, Fisher’s exact tests for categorical variables. Inter-observer agreement on the presence of LCD, PICC, PLS and PRS was calculated by Cohen’s kappa coefficients.

Univariable logistic regression was initially performed to assess the association of each ONH abnormality with the following explanatory variables: gender, age, AXL, MD, cpRNFLT, IOP and BMO area. Variables with a *p*-value < 0.2 in the univariable analysis were then included in a multivariable logistic regression model. A backward stepwise selection method, using a likelihood ratio test, was employed to build the final multivariable model, with adjustment for gender, age, and AXL. Results that remained significant after Bonferroni’s correction for multiple comparisons based on the number of included explanatory variables were discussed as the main findings of this study.

## Results

Data were collected from 686 eyes of 363 POAG subjects that met the inclusion criteria. After excluding 79 eyes due to glaucoma subtypes other than POAG, 8 eyes due to a diagnosis of pathological myopia, 3 eyes due to prior history of intraocular surgery and 110 eyes due to poor quality OCT or VF results and randomly selecting one eye of each subject, 263 eyes of 263 POAG eyes were enrolled in the study. Inter-examiner agreement was almost perfect for PRS and PICC (Cohen’s κ = 0.949 and 0.833, respectively), moderate for LCD (Cohen’s κ = 0.414), and substantial for PLS (Cohen’s κ = 0.734).

Baseline characteristics of the POAG eyes and comparison between eyes with and without HM are presented in Table 1. POAG eyes with HM had a higher proportion of male subjects, longer AXL, thicker cpRNFLT, thinner cpChT, larger BMO area and greater SFO/BMO offset magnitude in comparison with POAG eyes without HM. (*p* = 0.003, <0.001, <0.001, <0.001, <0.001, and <0.001 respectively) Comparison of baseline characteristics between eyes with and without ONH abnormality are presented in Supplementary Table S1 and S2.

**Table 1.** Baseline characteristics

	All OAG eyes *N* = 263	OAG eyes without HM *N* = 127	OAG eyes with HM *N* = 136	*p*-value
Gender (female)	155 (58.9%)	88 (69.3%)	67 (49.3%)	0.003^a^
Laterality (right)	127 (48.3%)	65 (51.2%)	62 (45.6%)	0.389^a^
Age (years)	60.3±9.6	60.4±9.2	60.2±10.0	0.840^b^
IOP (mmHg)	13.8±2.3	13.8±2.2	13.8±2.4	0.880^b^
CCT (μm)	521.2±37.2	519.2±38.6	522.8±36.1	0.558^b^
AXL (mm)	26.12±1.62	24.78±0.89	27.36±1.06	<0.001^b^
MD (dB)	-6.25±5.41	-5.74±4.51	-6.74±4.87	0.137^b^
cpRNFLT (μm)	76.6±12.2	73.0±11.2	79.9±12.3	<0.001^b^
cpChT (μm)	123.0±55.0	145.4±59.8	101.9±39.9	<0.001^c^
BMO area (mm^2^)	2.71±1.03	2.15±0.56	3.25±1.10	<0.001^c^
SFO/BMO offset magnitude (μm)	373.6±217.1	255.2±172.0	484.1±195.9	<0.001^b^

*OAG* open angle glaucoma, *HM* high myopia, *IOP* intraocular pressure, *CCT* central corneal thickness, *AXL* axial length, *MD* mean deviation, *cpRNFLT* circumpapillary retinal nerve fiber layer thickness, *cpChT* circumpapillary choroidal thickness, *BMO* Bruch’s membrane opening, *SFO* scleral flange opening.

Expressed as mean±SD or N(%)

*p*-values for comparison between OAG eyes with and without HM.

^a^Chi-square test

^b^Unpaired t-test

^c^Welch’s t-test

**Fig. 6:**
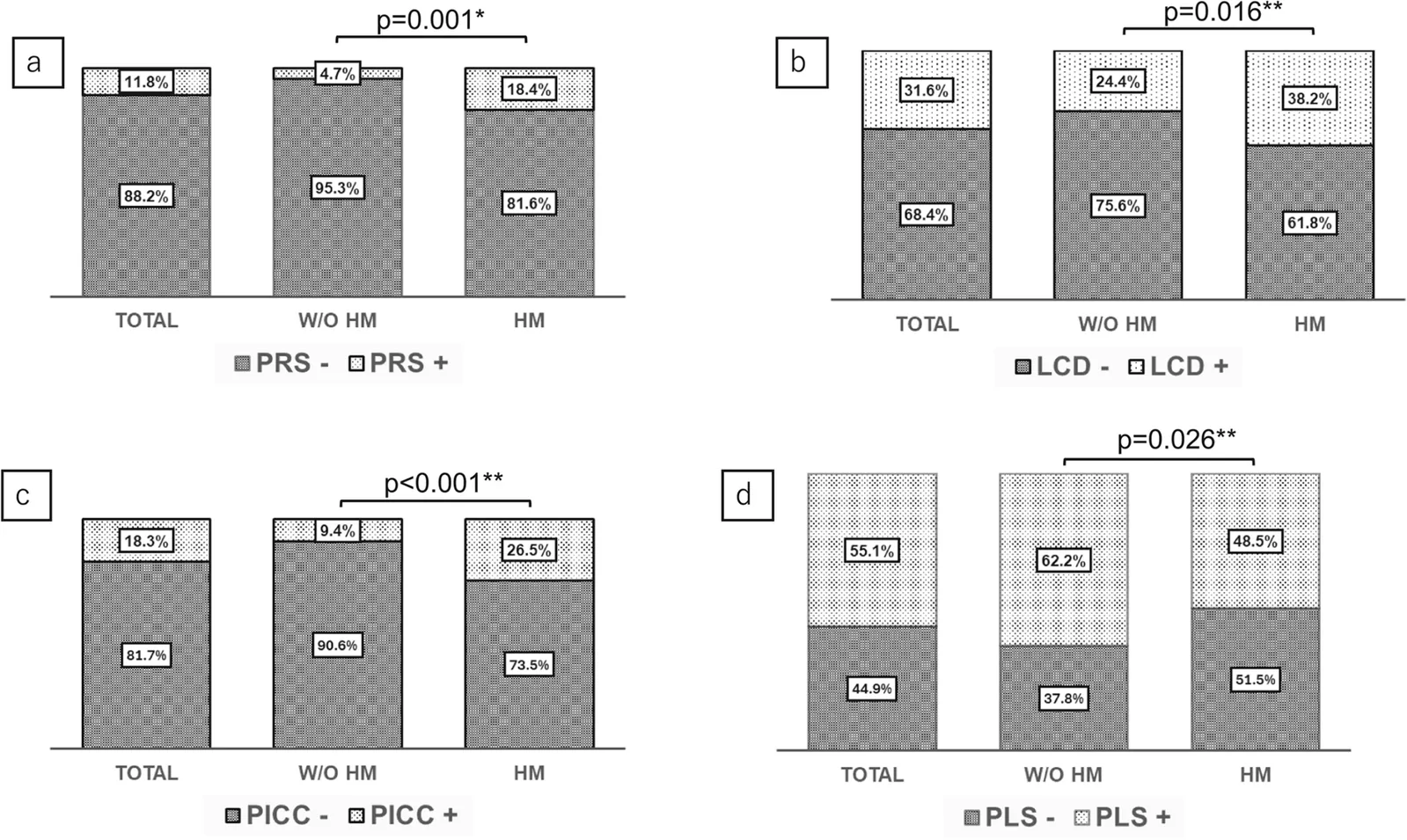
Frequency of optic nerve head abnormalities. **a** PRS: peripapillary retinoschisis; HM: high myopia; w/o: without. PRS was more frequent in eyes with HM (*p* = 0.001). **b** *LCD* lamina cribrosa defect, *HM* high myopia. LCD was more frequent in eyes with HM (*p* = 0.016). **c** *PICC* peripapillary intrachoroidal cavitation; *HM* high myopia. PICC was more frequent in eyes with HM (*p* < 0.001). **d** *PLS* prelaminar schisis, *HM* high myopia. PLS was more frequent in eyes without HM (*p* = 0.026). *Fisher’s exact test. **Chi-square test

PRS, LCD, PICC and PLS were present in 11.8%, 31.6%,18.3% and 55.1% of all POAG eyes respectively (Fig. 6). Within the LCDs, peripheral disinsertion was the most frequently observed (79.0%) followed by central defects (12.0%) and acquired pit-like defects (9.0%). Average LCD width was 176.30 ± 46.46 μm (range: 105.37–338.59). At least one type of ONH abnormality was observed in 77.2% of the eyes. 46.0%, 24.0%, 6.1% of the eyes presented 1, 2 and 3 ONH abnormalities, respectively. 1.1% of the eyes presented all 4 ONH abnormalities. (Table 2)

**Table 2:** Coexistence of optic nerve head abnormality

		N (%) total=263
No ONH abnormality		60 (22.8%)
1 ONH abnormality (46.0%)	PRS	7 (2.6%)
	LCD	28 (10.6%)
	PICC	5 (1.90%)
	PLS	81 (30.8%)
2 ONH abnormalities (24.0%)	PRS &LCD	2 (0.76%)
	PRS & PICC	1 (1.14%)
	PRS & PLS	10 (3.8%)
	LCD & PICC	14 (5.3%)
	LCD & PLS	24 (9.1%)
	PICC & PLS	12 (4.5%)
3 ONH abnormalities (6.1%)	PRS & LCD & PICC	1 (0.4%)
	PRS & LCD & PLS	3 (1.1%)
	PRS & PICC & PLS	4 (1.5%)
	LCD & PICC & PLS	8 (3.0%)
4 ONH abnormalities	LCD & PICC & PLS & PRS	3 (1.1%)

*ONH* optic nerve head, *PRS* peripapillary retinoschisis, *LCD* lamina cribrosa defect, *PICC* peripapillary intrachoroidal cavitation, *PLS* prelaminar schisis

PRS, LCD and PICC were observed more frequently in POAG eyes with HM (*p* = 0.001, 0.016, <0.001, respectively) while PLS was observed more frequently in POAG eyes without HM (*p* = 0.026) (Fig. 6).

The presence of PRS was associated with longer AXL in both univariable and multivariable analysis (both *p* < 0.001). The presence of LCD was associated with longer AXL and larger BMO area in univariable analysis but only BMO area (*p* = 0.009) remained statistically significant in the multivariable analysis. The presence of PICC was associated with longer AXL, thicker cpRNFLT and larger BMO area in univariable analysis but only larger BMO area remained significant in the multivariable analysis after correction for multiple comparison. (*p* < 0.001). The presence of PLS was associated with shorter AXL in both univariable and multivariable analyses. (*p* = 0.005) (Tables 3, 4, 5, 6)

**Table 3.** Univariable and multivariable logistic regression analysis for factors associated with presence of peripapillary retinoschisis

	Univariable analysis		Multivariable analysis	
	Odds ratio (95%CI)	*p*-value	Odds ratio (95%CI)	*p*-value
Gender (M/F)	1.079 (0.500, 2.326)	0.847		
Age (years)	1.023 (0.983, 1.065)	0.257		
AXL (mm)	1.652 (1.291, 2.113)	<0.001	1.686 (1.311, 2.170)	<0.001
MD (dB)	1.037 (0.961, 1.120)	0.347		
cpRNFLT (μm)	1.032 (1.000, 1.065)	0.050		
IOP (mmHg)	1.101 (0.943, 1.285)	0.223		
BMO area (mm^2^)	1.360 (0.986, 1.876)	0.061		

*AXL* axial length, *M* male, *F* female, *MD* mean deviation, *cpRNFLT* circumpapillary retinal nerve fiber layer thickness, *IOP* intraocular pressure at time of image acquisition, *BMO* Bruch’s membrane opening

Multivariable analysis conducted with backward stepwise method with likelihood ratio

Bold letters: statistically significant p-values after Bonferroni’s correction in multivariable analysis

**Table 4.** Univariable and multivariable logistic regression analysis for factors associated with presence of lamina cribrosa defect

	Univariable analysis		Multivariable analysis	
	Odds ratio (95%CI)	*p*-value	Odds ratio (95%CI)	*p*-value
Gender (M/F)	0.773 (0.456,1.309)	0.338		
Age (years)	0.984 (0.958,1.011)	0.249		
AXL (mm)	1.443 (1.210, 1.720)	<0.001	1.212 (0.963, 1.526)	0.102
MD (dB)	0.970 (0.925, 1.017)	0.205		
cpRNFLT (μm)	1.005 (0.984, 1.027)	0.649		
IOP (mmHg)	0.932 (0.831, 1.045)	0.228		
BMO area (mm^2^)	1.908 (1.454, 2.504)	<0.001	1.585 (1.124, 2.235)	0.009

*AXL* axial length, *M* male, *F* female, *MD* mean deviation, *cpRNFLT* circumpapillary retinal nerve fiber layer thickness, *IOP* intraocular pressure at time of image acquisition, *BMO* Bruch’s membrane opening

Multivariable analysis conducted with backward stepwise method with likelihood ratio

Bold letters: statistically significant *p*-values after Bonferroni’s correction in multivariable analysis

**Table 5.** Univariable and multivariable logistic regression analysis for factors associated with presence of peripapillary intrachoroidal cavitation

	Univariable analysis		Multivariable analysis^1^	
	Odds ratio (95%CI)	*p*-value	Odds ratio (95%CI)	*p*-value
Gender (M/F)	0.758 (0.403, 1.423)	0.389		
Age (years)	1.005 (0.972, 1.038)	0.787		
AXL (mm)	1.615 (1.305, 1.998)	<0.001		
MD (dB)	1.054 (0.987, 1.126)	0.116	1.085 (1.003, 1.175)	0.043
IOP (mmHg)	0.977 (0.853, 1.119)	0.738		
cpRNFLT (μm)	1.032 (1.005, 1.060)	0.021		
BMO area (mm^2^)	3.104 (2.189, 4.400)	<0.001	3.391 (2.340, 4.913)	<0.001

*AXL* axial length, *M* male, *F* female, *MD*: mean deviation, *cpRNFLT* circumpapillary retinal nerve fiber layer thickness, *IOP* intraocular pressure at time of image acquisition, *BMO* Bruch’s membrane opening

Multivariable analysis conducted with backward stepwise method with likelihood ratio

Bold letters: statistically significant p-values after Bonferroni’s correction in multivariable analysis

**Table 6.** Univariable and multivariable logistic regression analysis for factors associated with presence of prelaminar schisis

	Univariable analysis		Multivariable analysis	
	Odds ratio (95%CI)	*p*-value	Odds ratio (95%CI)	*p*-value
Gender (M/F)	1.169 (0.712, 1.917)	0.537		
Age (years)	1.028 (1.002, 1.054)	0.037	1.029 (1.002, 1.056)	0.035
AXL (mm)	0.791 (0.676, 0.926)	0.004	0.795 (0.677, 0.934)	0.005
MD (dB)	0.993 (0.949, 1.038)	0.744		
cpRNFLT (μm)	0.998 (0.978, 1.018)	0.814		
IOP (mmHg)	1.095 (0.984, 1.219)	0.095	1.112 (0.996, 1.241)	0.058
BMO area (mm^2^)	0.997 (0.787, 1.264)	0.982		

*AXL* axial length, *M* male, *F* female, *MD* mean deviation, *cpRNFLT* circumpapillary retinal nerve fiber layer thickness, *IOP* intraocular pressure at time of image acquisition, *BMO* Bruch’s membrane opening

Multivariable analysis conducted with backward stepwise method with likelihood ratio

Bold letters: statistically significant *p*-values after Bonferroni’s correction in multivariable analysis

## Discussion

Our study found that 77.2% of POAG eyes exhibited at least one ONH abnormality, with considerable overlap among them. PLS was the most prevalent abnormality, followed by LCD, PICC, and PRS. PRS, LCD and PICC were more frequently observed in eyes with HM and were significantly associated with longer AXL and/or larger BMO. Conversely, PLS was more frequent in eyes without HM and was associated with shorter AXL.

Increasing AXL leads to ocular tissue stretching and remodeling in myopic eyes. When biomechanical stress exceeds the tissue's adaptive and remodeling capacity, tissue splitting, tearing, and rupturing may occur [11], potentially leading to ONH abnormalities such as PRS, LCD, PICC, and PLS. ONH abnormalities were common in our myopic POAG cohort, with 31.2% of eyes exhibiting more than two types. The prevalence of PRS (11.8%) was higher than previously reported in POAG eyes (1.5-6.0%) [9, 31–33], most likely due to the longer average AXL in our cohort. While most PRS cases were limited to the inner retinal layers (nerve fiber layer, ganglion cell layer, inner plexiform layer), five eyes showed PRS extending to the inner nuclear layer, outer plexiform layer or outer nuclear layer. LCD was observed in 31.6% of eyes, comparable to other studies of POAG eyes [14, 16, 34], although some report higher frequencies (75-90%) [12, 13]. Earlier studies with lower LC visibility due to older OCT technology may have inadvertently included large LC pores as LCDs. Our average LCD width of 176.30± 46.46μm aligns with prior reports [12, 15]. However, Xie et al. found LCDs up to 800μm wide in pathologically myopic eyes [23], suggesting stronger ONH deformation in eyes with extensive myopic changes. Peripheral disinsertion was the most common LCD subtype. PICC was observed in 18.3% of eyes. Although the frequency of PICC in a general POAG cohort has not been previously reported, our findings exceed the 17% prevalence found in a population-based cohort of HM eyes [35]. This may be attributable to our focus on POAG eyes, potentially indicating a higher prevalence of PICC in this population. PLS was seen in 55.1% of POAG eyes, a frequency comparable to previous reports in POAG eyes [19, 20, 22].

Our study reveals significant associations between AXL and ONH abnormalities. While retinal splitting in the peripapillary region, similar to myopia-related macular retinoschisis, has been observed in HM eyes [8, 23], the relationship between AXL and PRS in POAG eyes with a wide range of AXLs had not been previously reported. We found that PRS occurred more frequently in HM eyes and that longer AXL was associated with PRS in both univariable and multivariable analyses. Consistent with our findings, Jiang et al. report longer AXL in HM eyes with PRS [8], and Nishijima et al. found an association between longer AXL and extensive PRS extending into the ONH [33]. Mechanistically, PRS limited to the inner retinal layers is thought to be caused by posterior vitreous traction creating fissures between the inner limiting membrane (ILM) and the nerve fiber layer or within the nerve fiber layer. On the other hand, PRS of the outer retinal layers may involve mechanical stress arising from differential responses of the retina and the sclera to AXL elongation, similar to macular retinoschisis [36]. In our cohort, 4 of the 5 eyes with PRS extending to the outer retinal layers had an AXL greater than 26 mm, suggesting a stronger association with HM. However, due to the small number of such cases, this observation was descriptive and not subjected to formal statistical analysis.

The association of LCD and AXL is controversial in previous studies. While several studies report no relationship between longer AXL and LCD presence [8, 13, 14, 23], two reports from Korea found a significant association in multivariable analyses [15, 16]. Consistent with these reports, our results also show that LCD was more frequent in HM eyes and that longer AXL was associated with LCD in univariable analysis. This may be partially due to the varying etiologies and clinical significance of LCD depending on its location. Evidence suggests that myopic eyes are more prone to peripheral disinsertions compared to other LCD subtypes [37]. Myopia-related remodeling, including misalignment of retinal, choroidal, and scleral layers along with neural canal expansion [38, 39], exerts horizontal shearing stress on the LC, potentially leading to its disinsertion from its scleral anchor. In support of this, all peripheral disinsertions observed in our cohort were located in the temporal region and had significantly longer average AXL compared to other LCD subtypes (26.91±1.58mm vs. 26.08±0.31mm, p=0.034). The high proportion (79%) of peripheral disinsertions observed in our cohort may have strengthened the observed association between AXL and LCD.

The association of PICC and HM is well documented [8, 23, 35]. Our results confirm that PICC was more frequent in HM eyes and demonstrate significant association with longer AXL in univariable analysis. Previous studies propose that AXL elongation associated border tissue rupture is a key factor in PICC formation [40]. Extensive myopic ONH structural changes, particularly in the temporal region, lead to elongation of the border tissues of Jacoby and Elschnig. These border tissues connecting the BMO and LC play a biomechanically important role in LC stabilization [41] and disruption of these tissues can compromise their structural integrity.

However, PRS, LCD and PICC were also observed in eyes without HM. This agrees with recent reports from our group indicating that structural deformations accompanying AXL elongation may play a more critical role in PICC formation than AXL itself [26]. Supporting this, our multivariable analysis demonstrates that larger BMO area was more strongly associated with the presence of LCD and PICC. This suggests that biomechanical stress resulting from expansion of the neural canal is an independent factor contributing to ONH abnormalities.

On the other hand, PLS was more frequent in POAG eyes without HM and shorter AXL was associated with the presence of PLS in both univariable and multivariable analysis. This aligns with a previous study in a cohort of advanced POAG subjects [20]. While those authors suggest stronger posterior vitreous traction in eyes with shorter AXL due to incomplete posterior vitreous detachment, we found no association between posterior vitreous detachment grading and PLS in another study using a different dataset from our current study [21]. Previous studies associating deeper cupping to PLS suggest that PLS development involves the detachment of blood vessels and the intra-disc ILM/meniscus of Kuhnt from the underlying prelaminar tissue, as the optic disc excavates in glaucoma [19]. Since the LC is known to become shallower in eyes with longer AXL [39], optic disc cup deepening may have been diminished in POAG eyes with HM due to the combined effect of myopia and glaucoma. However, our results should be interpreted with caution, as reduced visibility in the nasal region of HM eyes may have led to the underdetection of PLS. This limitation is less likely to affect the detection of peripapillary or central ONH abnormalities, such as PRS, LCD, and PICC, but may hinder identification of PLS, which often occurs along the lateral margins of the ONH cup. Although this limitation applies to all studies evaluating PLS, its impact may have been more pronounced in the present study due to the inclusion of eyes with longer axial lengths compared with prior reports [19–21].

Of note, the presence of PRS, LCD and PLS did not demonstrate significant association with worse 24-2 MD even though these ONH abnormalities have all been associated with glaucoma [8–10, 12, 13, 15–17, 19, 22, 23, 31, 33]. The extensive overlap among ONH abnormalities, as seen from our results, made it difficult to attribute glaucomatous damage to any single structural change. PRS is known to be transient. A longitudinal study reports no immediate impact of de novo PRS on 24-2 MD or VF sensitivity [42], suggesting a non-simultaneous relationship between PRS and VF. The correspondence between VF damage and LCD varies with LCD location, with the best correspondence seen in inferior temporal LCDs [37]. Furthermore, we recently reported that approximately half of PICCs do not present location-specific VF damage [17], indicating that these ONH abnormalities alone do not necessarily contribute to detectable VF damage. The association between PLS and glaucoma also remains controversial. We previously reported that there was no difference in PLS frequency between age- and AXL-matched healthy and glaucomatous eyes (79.1% vs. 72%), nor any association between PLS and VF indices in another dataset [21]. The high prevalence of myopia in Asian POAG eyes [43] may mitigate glaucomatous cup deepening due to LC shallowing associated with AXL elongation, potentially altering the relationship between PLS and glaucoma.

Some limitations need to be acknowledged in our study. First, the use of radial scans may have limited our ability to detect smaller ONH changes. However, we also examined ONH cube scans to ensure that we did not miss large or clinically significant findings. Second, we could not evaluate PPA-beta because our current software could not reliably identify the retinal pigment epithelium (RPE) termination in highly myopic eyes, in which border tissue elongation often makes the RPE edge ambiguous. However, because the primary aim of this study was to evaluate the relationship between ONH abnormalities and AXL, we expect that the lack of PPA-beta analysis had limited impact on our AXL–focused results, as PPA-beta is more strongly associated with aging and glaucomatous remodeling than with axial elongation. [44, 45] Future studies are needed to evaluate the relationship between PPA-beta, ONH abnormalities, and glaucomatous damage. Third, the cross-sectional design prevents us from exploring the temporal relationship between AXL elongation and the development of these ONH abnormalities. The association of larger BMO area with LCD and PICC suggests that BMO expansion during myopia development may contribute to these structural disruptions. Future longitudinal studies in younger patients are necessary to address this issue. Fourth, this study did not include a non-glaucomatous control group examined using the same OCT device and acquisition protocol. Although comparisons with healthy eyes are important for clarifying glaucoma-specific features of AXL–related ONH abnormalities, such direct comparisons were not feasible due to differences in imaging platforms and study frameworks across datasets. Future studies using unified imaging platforms and parallel control cohorts are warranted. Fifth, our study cohort was limited to Japanese eyes, and our results may not be compatible with POAG cohorts of other races. Racial differences in deep ONH structures are well-known [46, 47] and AXL elongation may have varying impacts on structural abnormalities across different racial groups.

In conclusion, ONH abnormality was common in myopic POAG eyes with a large overlap. The presence of PRS, LCD and PICC were associated with longer AXL and myopic ONH structure changes while PLS was associated with shorter AXL. Understanding the relationship between myopia and these ONH abnormalities is essential for elucidating the structural changes that occur with AXL elongation and will provide a foundation for future studies exploring the functional significance of these abnormalities.

## Supplementary Information

Below is the link to the electronic supplementary material.

Supplementary file1 (PPTX 1075 KB) Figure. Identification of deep optic nerve head structures.[17]. a. Bruch’s membrane opening (BMO) (orange dots) and scleral flange opening (SFO) (blue dots) were determined upon 12 radial optical coherence tomography B-scans. b. BMO and SFO points are overlayed upon the line scanning laser ophthalmoscopy image. The temporal area between BMO and SFO contains peripapillary zone without Bruch’s membrane and a region of exposed scleral flange (yellow area). c. SFO/BMO offset magnitude was defined as the distance between BMO centroid and SFO centroid projected onto the BMO plane (red line).

Supplementary file2 (DOCX 20 KB)
