# Supplementary material for: Optic nerve head abnormalities in primary open-angle glaucoma and their associations with axial length
Source: Jpn J Ophthalmol. 2026 May 8;70(5):1229–40. doi: 10.1007/s10384-026-01371-y (PMC13615988; doi:10.1007/s10384-026-01371-y)
Supplement: Supplementary file 1 [file 10384_2026_1371_MOESM2_ESM.docx]

Supplementary Table 1. Comparison of baseline characteristics between eyes with and without peripapillary retinoschisis and lamina cribrosa defect

|  | PRS- N=232 | PRS+  N=31 | p-value | LCD-  N=180 | LCD+ N=83 | *p*-value |
| --- | --- | --- | --- | --- | --- | --- |
| Gender (female) | 138 (59.5%) | 19 (61.3%) | 0.847† | 111 (61.7%) | 46 (55.4%) | 0.337† |
| Laterality (right) | 112 (48.3%) | 15 (48.4%) | 0.991† | 89 (49.4%) | 38 (45.8%) | 0.581† |
| Age (years) | 60.0±9.7 | 62.1±9. | 0.257* | 60.8±9.2 | 59.3±10.6 | 0.250* |
| IOP (mmHg) | 13.8±2.2 | 14.3±3.0 | 0.223* | 13.9±2.2 | 13.6±2.6 | 0.254** |
| AXL (mm) | 25.96±1.55 | 27.26±1.69 | <0.001* | 25.83±1.58 | 26.74±1.55 | <0.001* |
| MD (dB) | -6.37±5.53 | -5.39±4.43 | 0.347* | -5.97±5.23 | -6.88±5.77 | 0.204* |
| cpRNFLT (μm) | 76.0±12.4 | 80.7±10.1 | 0.045* | 76.3±12.2 | 77.1±12.3 | 0.651* |
| cpChT (μm) | 126.6±55.6 | 94.7±40.3 | 0.003* | 126.7±57.5 | 114.6±48.1 | 0.100* |
| BMO area (mm^2^) | 2.67±1.02 | 3.05±1.11 | 0.057* | 2.50±0.86 | 3.19±1.22 | <0.001** |
| SFO/BMO offset magnitude (μm) | 358.1±210.7 | 488.8±232.1 | 0.002* | 321.0±204.1 | 488.4±200.6 | <0.001* |

PRS: peripapillary retinoschisis; LCD: lamina cribrosa defect; IOP: intraocular pressure; AXL: axial length; MD: mean deviation; cpRNFLT: circumpapillary retinal nerve fiber layer thickness; cpChT: circumpapillary choroidal thickness; BMO: Bruch’s membrane opening; SFO: scleral flange opening.

Expressed as mean±SD or N(%)

*p*-values for comparison between eyes with and without ONH abnormality.

†: Chi-square test

*: Unpaired t-test

**:Welch’s t-test

Supplementary Table 2. Comparison of baseline characteristics between eyes with and without peripapillary intrachoroidal cavitation and prelaminar schisis

|  | PICC- N=215 | PICC+  N=48 | p-value | PLS-  N=118 | PLS+ N=145 | *p*-value |
| --- | --- | --- | --- | --- | --- | --- |
| Gender (female) | 131 (60.9%) | 26 (54.2%) | 0.388† | 68 (57.6%) | 89 (61.4%) | 0.537† |
| Laterality (right) | 109 (50.7%) | 18 (37.5%) | 0.098† | 67 (56.8%) | 69 (47.6%) | 0.138† |
| Age (years) | 60.2±9.4 | 60.6±10.8 | 0.788* | 58.9±9.3 | 61.4±9.8 | 0.036* |
| IOP (mmHg) | 13.8±2.3 | 13.7±2.4 | 0.739* | 13.5±2.3 | 14.0±2.3 | 0.094* |
| AXL (mm) | 25.90±1.56 | 27.10±1.54 | <0.001* | 26.44±1.47 | 25.85±1.70 | 0.003* |
| MD (dB) | -6.50±5.67 | -5.14±3.95 | 0.050** | -6.13±5.21 | -6.35±5.59 | 0.745* |
| cpRNFLT (μm) | 75.8±12.3 | 80.3±11.3 | 0.019* | 76.8±11.9 | 76.4±12.5 | 0.814* |
| cpChT (μm) | 127.3±57.1 | 103.1±38.4 | 0.001** | 125.8±56.5 | 120.6±53.8 | 0.443* |
| BMO area (mm^2^) | 2.48±0.82 | 3.76±1.24 | <0.001* | 2.72±1.08 | 2.71±1.00 | 0.982** |
| SFO/BMO offset magnitude (μm) | 328.6±195.6 | 578.5±192.2 | <0.001** | 422.4±202.8 | 333.3±220.9 | 0.001* |

PICC: peripapillary intrachoroidal cavitation; PLS: prelaminar schisis; IOP: intraocular pressure; AXL: axial length; MD: mean deviation; cpRNFLT: circumpapillary retinal nerve fiber layer thickness; cpChT: circumpapillary choroidal thickness; BMO: Bruch’s membrane opening; SFO: scleral flange opening.

Expressed as mean±SD or N(%)

*p*-values for comparison between eyes with and without ONH abnormality.

†: Chi-square test

*: Unpaired t-test

**:Welch’s t-test
